# Non-Linear Lymphatic Anatomy in Breast Cancer Patients Prior to Axillary Lymph Node Dissection: A Risk Factor For Lymphedema Development

**DOI:** 10.1007/s10911-023-09545-x

**Published:** 2023-07-22

**Authors:** JacqueLyn R. Kinney, Rosie Friedman, Erin Kim, Elizabeth Tillotson, Kathy Shillue, Bernard T. Lee, Dhruv Singhal

**Affiliations:** Division of Plastic and Reconstructive Surgery, Beth Israel Deaconess Medical Center, Harvard Medical School, 110 Francis St, Suite 5A, BostonBoston, MA 02215 USA

**Keywords:** Breast cancer related lymphedema, Immediate lymphatic reconstruction, Prevention, Trauma, Lymph node biopsy, Lymphatic anatomy

## Abstract

Immediate lymphatic reconstruction (ILR) at the time of axillary lymph node dissection (ALND) has become increasingly utilized for the prevention of breast cancer related lymphedema. Preoperative indocyanine green (ICG) lymphography is routinely performed prior to an ILR procedure to characterize baseline lymphatic anatomy of the upper extremity. While most patients have linear lymphatic channels visualized on ICG, representing a non-diseased state, some patients demonstrate non-linear patterns. This study aims to determine potential inciting factors that help explain why some patients have non-linear patterns, and what these patterns represent regarding the relative risk of developing postoperative breast cancer related lymphedema in this population. A retrospective review was conducted to identify breast cancer patients who underwent successful ILR with preoperative ICG at our institution from November 2017—June 2022. Among the 248 patients who were identified, 13 (5%) had preoperative non-linear lymphatic anatomy. A history of trauma or surgery of the affected limb and an increasing number of sentinel lymph nodes removed prior to ALND appeared to be risk factors for non-linear lymphatic anatomy. Furthermore, non-linear anatomy in the limb of interest was associated with an increased risk of postoperative lymphedema development. Overall, non-linear lymphatic anatomy on pre-operative ICG lymphography appears to be a risk factor for developing ipsilateral breast cancer-related lymphedema. Guided by the study’s findings, when breast cancer patients present with baseline non-linear lymphatic anatomy, our institution has implemented a protocol of prophylactically prescribing compression sleeves immediately following ALND.

## Introduction

**Fig. 1:**
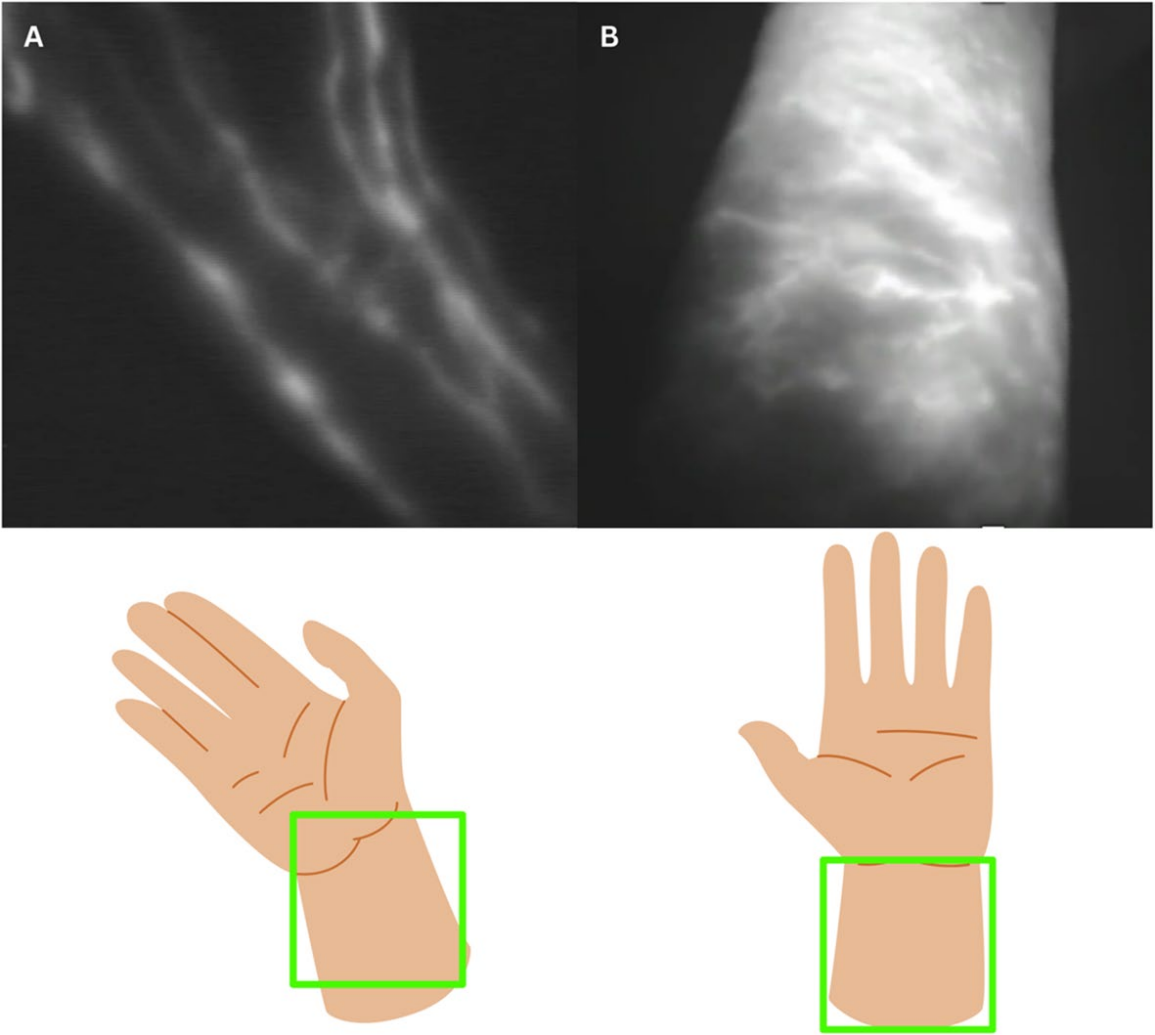
Linear and Non-Linear Lymphatic Anatomy Demonstrated Immediately Prior to Axillary Lymph Node Dissection with Pre-operative Indocyanine Green (ICG) Lymphography. **A** Linear lymphatic anatomy on ICG lymphography, **B** Non-linear lymphatic anatomy on ICG lymphography. Note: Figures were obtained from patients treated at the sponsoring institution. Permissions to utilize their study contents for the purpose of research and publication were obtained. Animated images were developed for the use of this study using Canva, an online design software

Immediate lymphatic reconstruction (ILR) has become increasingly recognized and adopted by lymphatic microsurgeons for the prevention of breast cancer related lymphedema. ILR involves anastomosing divided arm lymphatic channels to an adjacent vein at the time of axillary lymph node dissection (ALND), thereby restoring a pathway for lymphatic drainage [1]. As part of the preoperative evaluation prior to ILR, indocyanine green (ICG) lymphography of the upper extremities is utilized for visualization and anatomical mapping of a patient’s superficial lymphatic system [2–7]. Mapping of the lymphatic anatomy is done as part of our standard of care in order to (1) document an individual’s baseline anatomy and (2) serve as a potential guide for ICG-guided manual lymphatic drainage (MLD) or other therapies if the patient were to develop lymphedema. Multiple morphologies of lymphatic drainage on ICG lymphography have been described in the literature, including both linear and non-linear lymphatic channels (Fig. 1) [8] Linear channels are characterized by longitudinal fluorescent lines and typically reflect healthy lymphatic collectors [8–10]. In contrast, non-linear morphologies represent pathologic lymphatic obstruction and are associated with more severe stages of lymphedema [9, 11–13]. Interestingly, a small subset of breast cancer patients without prior history of lymphatic disease have been noted to have non-linear lymphatic anatomy at a baseline state, prior to undergoing ALND [3, 14]. The potential implications of these findings on lymphedema risk after ALND have not been previously studied.

**Fig. 2:**
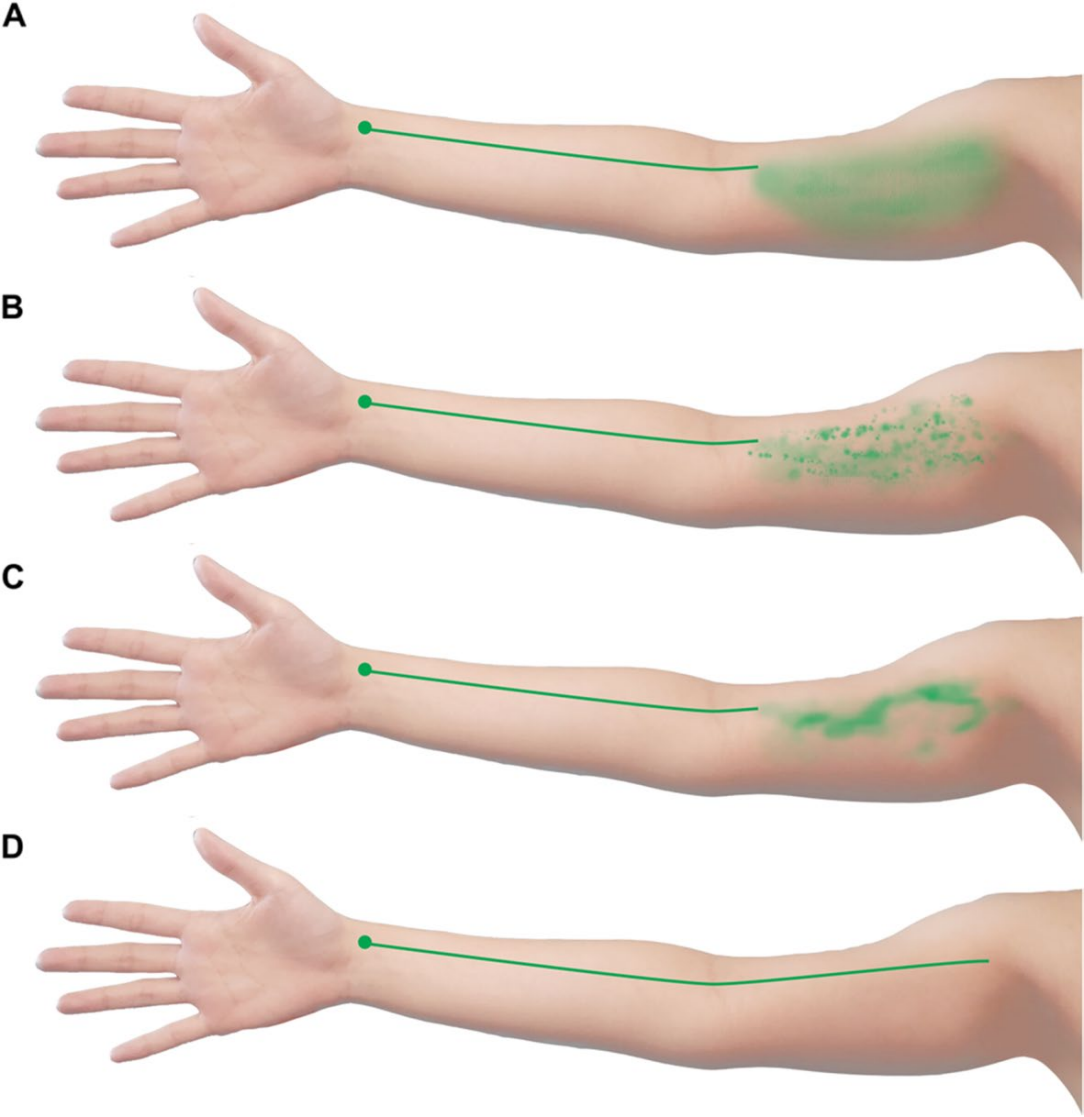
Demonstration of the Various Types of Upper Extremity Lymphatic Drainage as it would be Visualized with Indocyanine Green (ICG) Lymphography. **A** Diffuse patterning, **B** Stardust patterning, **C** Splash patterning, **D** Linear lymphatic patterning. Note: Figures were created for the use of this publication in Adobe Photoshop

Within the subset of non-linear morphology, also referred to as dermal backflow (DBF), the lymphatic drainage can be further differentiated as splash, stardust, or diffuse patterns (Fig. 2) [8]. Prior studies have suggested that with increasing severity of lymphedema, DBF patterns will follow a sequential evolution from splash to stardust to diffuse patterning [15, 16]. Prior studies on non-linear lymphatic anatomy have been particularly useful for developing methods for the early detection of lymphedema as well as for staging and grading the disease severity [15, 16]. However, investigations to date have not yet examined the implications of non-linear morphologies present in patients without lymphedema. Therefore, the effects of baseline non-linear morphologies on the risk of lymphedema development after ALND is unknown.

Recent studies have demonstrated that ILR can significantly decrease the risk of breast cancer related lymphedema in patients from a 33% incidence to a 9% incidence [1, 3, 16, 17]. However, the incidence of breast cancer related lymphedema among patients with preoperative non-linear anatomy is not well understood, including whether or not they receive the same benefit from ILR as breast cancer patients with linear anatomy. Therefore, this study aims to determine the incidence of breast cancer related lymphedema in patients with preoperative non-linear lymphatic anatomy in order to better understand the clinical significance and implications of these morphologies on patients’ risk of lymphedema.

## Methods

### Retrospective Review

A retrospective study was conducted at the Boston Lymphatic Center, Beth Israel Deaconess Medical Center. Institutional review board approval was obtained for this study (Protocol #2022P000450). A review of a prospectively maintained REDCap Quality Improvement Database [18] and electronic medical records was performed. Patients who received ICG lymphography prior to ALND and attempted ILR from November 2017 to June 2022 were identified. Patient demographics, prior medical and surgical history, operative records, and postoperative outcomes were extracted. A history of prior trauma to the limb of interest was recorded. Prior trauma was defined as having any injury, procedure, or surgical intervention on the upper limb undergoing the ICG lymphography, from the axillary region to the hand.

### ICG Lymphography

Prior to ALND, under sterile conditions, 0.1 cc of stock (2.5 mg/cc) ICG solution (Akorn Inc., Lake Forest, IL) mixed with 5% of albumin was injected intradermally. The locations of these injections were based on the lymphosome concept, and represent all the previously identified zones of the hand, forearm, and lateral upper arm pathway [2, 19]. By injecting each lymphosome, the major lymphatic drainage pathways of the hand and forearm are represented [19]. A near-infrared (NIR) imaging device, the PDE-Neo II (Hamamatsu Photonics KK, Hamamatsu, Japan) was used to visualize the superficial lymphatic channels of the extremity. All ICG lymphographies were performed by one of two members of the lymphatic surgery team (ET or DS) and interpreted by the same lymphatic surgeon (DS). The readings are recorded via a REDCap form for quantitative analysis of the patient population.

All ALNDs were performed by the same institutional general surgery team. After conclusion of ALND, ILR was attempted by the same lymphatic surgeon (DS). Patients in which ILR was intraoperatively aborted were excluded from the analysis. Following ILR, all patients entered our postoperative surveillance program for monitoring of lymphedema development as described in prior investigations [20]. A diagnosis of lymphedema was defined as the presence of symptoms consistent with lymphedema, such as pain, swelling, restricted range of motion, trouble fitting into clothing, feelings of heaviness or tightness, and one of the following objective findings: (1) relative limb volume change greater than 10% or (2) Lymphedema Index (L-Dex) greater than 10 from baseline. All measurements and physical exams were performed by a certified lymphedema therapist.

### Statistical Analysis

The data were separated into two cohorts based on whether patients had linear or non-linear findings on upper extremity ICG lymphography. To assess for differences between categorical variables, a Fisher exact test was performed. To assess for differences between continuous variables, Wilcoxon rank sum test was used. Multivariate logistic regression was utilized to assess the association between a history of trauma and the number of sentinel lymph nodes removed, with the odds of having a non-linear ICG lymphography finding. All analyses were conducted using R statistical programming language (version 4.2.1, R Core Team 2021, Vienna, Austria) and SAS Studio (Version 3.81, SAS Institute Inc 2013, Cary, NC, USA) [21]. Statistical significance was set at α = 0.05.

## Results

During the study period, a total of 275 patients had preoperative upper extremity ICG lymphography and ALND with an attempted ILR over the study period at our institution. Among these patients, 27 (10%) had an aborted ILR procedure and were excluded from the study. Towards the beginning of the study period, aborting the procedure was commonly due to inability to create the lymphovenous anastomosis under minimal tension. Since encorporating our new technique of utilizing a lower extremity vein graft to the anastamosis, aborted ILR procedures have been greatly decreased [22]. Notably, none of these patients had an aborted ILR due to lymphatic anatomy. Therefore, a total of 248 patients were included. The median age and body mass index (BMI) of the population was 57 years and 27 m/kg^2^ respectively. The vast majority of patients were female (97%). On preoperative ICG lymphography, 13 patients (5%) had preoperative non-linear lymphatic anatomy within the limb of interest. Demographics were similar between the linear and non-linear cohorts (Table 1).

**Table 1:** Population Characteristics

	**Total**	**Linear**	**Non-Linear**	***p*-value**
Total patients *n*	248	235 (95%)	13 (5%)	
Age *median (IQR)*	57 (49, 67)	57 (48, 67)	62 (53, 76)	0.26
**Gender *n (%)***				
Female	241 (97)	228 (97)	13 (100)	> 0.99
Male	6 (2)	6 (3)	0 (0)	
Unknown	1 (0)	1 (0)	0 (0)	
**Race *n (%)***				
White	177 (71)	168 (71)	9 (69)	0.70
Black or African American	28 (11)	26 (11)	2 (15)	
Asian	17 (7)	16 (7)	1 (8)	
Other	10 (4)	9 (4)	1 (8)	
Unknown	16 (6)	16 (7)	0 (0)	
**Ethnicity *n (%)***				
Hispanic or Latino	12 (5)	10 (4)	2 (15)	0.14
Not Hispanic or Latino	217 (88)	206 (88)	11 (85)	
Unknown	19 (8)	19 (8)	0 (0)	
**BMI**	27 (24, 32)	27 (24, 32)	28 (25, 34)	0.36

**Fig. 3:**
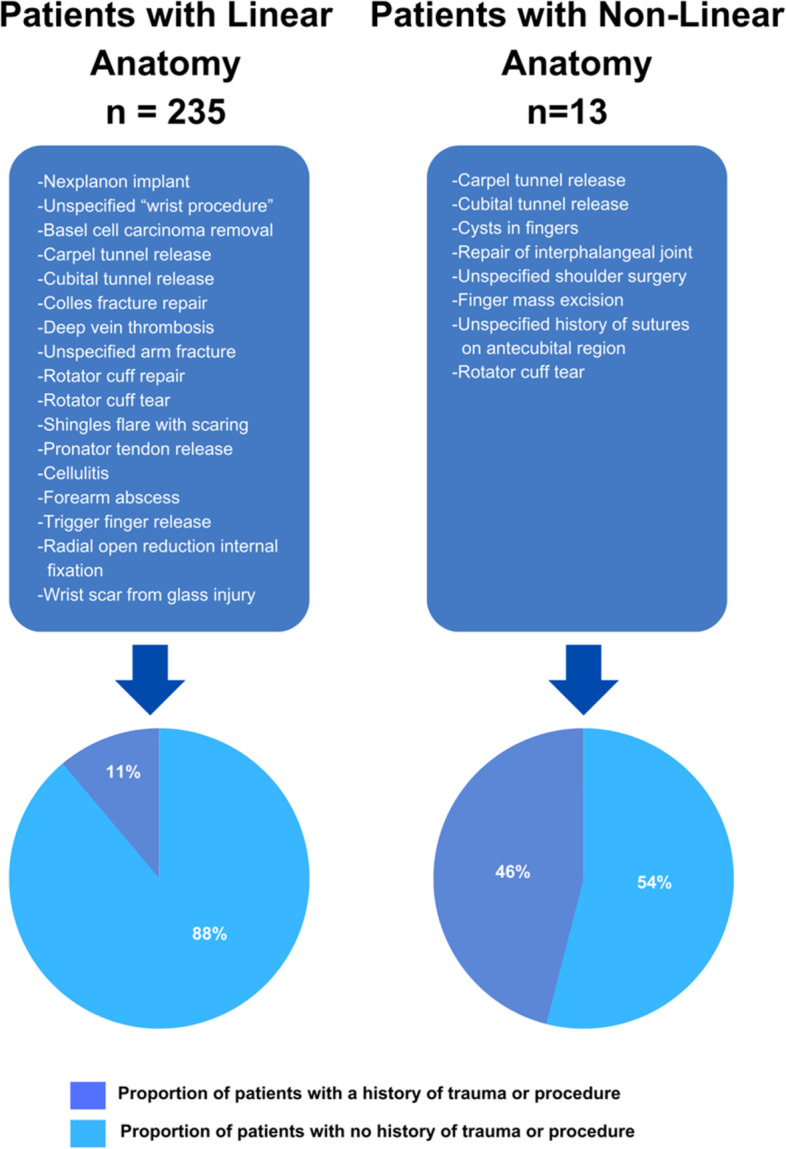
All Reported Trauma History in Patients Receiving Upper Extremity ICG Lymphography Prior to Ipsilateral Axillary Lymph Node Dissection. Note: Designed with Canva, an online software, for the purpose of this publication

When comparing the linear and non-linear cohorts, a greater proportion of the non-linear cohort had a history of trauma, injury, or invasive procedures (46%) to the upper extremity compared to the linear cohort (11%) (*p* = 0.004). A comprehensive list of upper extremity procedures, injuries, and traumas for each cohort is displayed in Fig. 3.

Regarding oncologic evaluation, the two cohorts had comparable rates of receiving sentinel lymph node biopsies (54% of the linear cohort compared to 46% of the non-linear cohort). Notably, the median number of sentinel lymph nodes removed was higher in the non-linear cohort (6) compared to the linear cohort (3) (Table 2). Logistic regression modeling demonstrated that the total number of sentinel lymph nodes removed was associated with 1.2 odds of having a non-linear anatomy (*p* = 0.02). Additionally, having trauma to the limb of interest was associated with 6.0 increased odds of having non-linear lymphatic anatomy (*p* = 0.020). The number of positive nodes removed during the sentinel lymph node biopsy, the number of nodes removed during the ALND, and the number of positive nodes removed during ALND were similar across the groups.

**Table 2:** Oncologic Characteristics of Breast Cancer Patients With Upper Extremity Non-linear Lymphatic Anatomy On Preoperative Indocyanine Green Lymphography

	**Total**	**Linear**	**Non-Linear**	***p*-value**
**Total patients *n***	248	235 (95%)	13 (5%)	
**SNLB** *n (%)*	131 (53%)	126 (54%)	6 (46%)	0.78
** Total nodes removed** * Median (IQR)*	3 (2, 5)	3 (2, 4)	6 (4, 11)	0.08
** Total positive nodes removed** * Median (IQR)*	2 (1, 3)	2 (1, 3)	4 (2, 5)	0.19
**ALND** *n (%)*	248 (100%)	235 (100%)	13 (100%)	
** Total nodes removed** * Median (IQR)*	17 (13, 23)	17 (13, 23)	19 (12, 23)	0.85
** Total nodes removed** * Median (IQR)*	1 (0, 3)	1 (0, 3)	1 (0, 2)	0.69
**Prior history of trauma or procedure on the affected limb** *n (%)*	32 (13%)	26 (11%)	6 (46%)	0.004

*SNLB* sentinel lymph node biopsy, *ALND* axillary lymph node dissection, *IQR* interquartile range

### Non-Linear Subgroup Analysis

The 13 patients with non-linear lymphatic anatomy were assessed for incidence of postoperative lymphedema development. All patients were female (100%) with an average age of 57.2 years (SD 10.8), and an average body mass index (BMI) of 31.9 kg/m^2^ (SD 6.1). All participants received adjuvant radiation (100%), 8 patients (67%) received neoadjuvant chemotherapy, and 8 patients (67%) received adjuvant chemotherapy. Regarding the postoperative incidence of lymphedema, one patient had neoadjuvant taxol-induced lymphedema prior to ILR and was therefore excluded from the investigation of lymphedema incidence. The remaining 12 patients reported no symptoms of lymphedema prior to ILR and were therefore included.

Of the 12 included patients, the total number of non-linear patients meeting clinical criteria for lymphedema was 5 (42%) and 4 (33%) did not. Notably, the remaining 3 (25%) patients did not meet all clinical criteria for lymphedema diagnosis, but showed signs of lymphedema with either increase in objective values (L-Dex > 10%, a relative volume change > 10% in the limb of interest) or had symptoms consistent with lymphedema. These patients were classified as having “sub-clinical lymphedema.” The average follow-up time was 10.8 months (SD 7.3). The average time from ILR to lymphedema diagnosis was 6.7 months (SD 3.0).

## Discussion

In this study, we investigated the implications of preoperative non-linear upper extremity lymphatic anatomy on the incidence of breast cancer-related lymphedema. Our findings demonstrate that patients with non-linear anatomy (1) had a higher rate of prior surgery or trauma to the limb, (2) a greater number of lymph nodes removed during preoperative sentinel lymph node biopsy, and (3) an increased incidence of lymphedema following ALND and ILR.

This study suggests a possible association between prior trauma or injury to the superficial lymphatic channels correlating with a higher risk of lymphedema following oncologic breast interventions. Understanding the association between the surgical treatment for breast cancer and the development of lymphedema is critical, as one in five patients develop upper extremity lymphedema after lymph node extraction and mastectomy [1, 23]. Our Lymphatic Center, a certified Center of Excellence, invests significant focus on researching surgical and non-invasive interventions associated with reduced rates of lymphedema, thereby allowing for robust investigations into this patient population [24]. Our center has previously observed that of the breast cancer patients who had received a successful ILR procedure after ALND by the same surgeon, 9% of the cohort went on to develop post-operative lymphedema after 11 months of followup [17]. Notably, in this study’s assessment of patients specifically with non-linear anatomy, 42% of patients developed a clinical lymphedema diagnosis with an additional 25% showing signs of lymphedema. Additionally, these patients had a shorter follow-up compared to those patients in the previous study (10.5 months compared to 17 months) [17]. As lymphedema can develop in a delayed fashion, having a larger incidence in breast cancer related lymphedema with a significantly shorter follow-up is likely to underestimate the incidence of breast cancer related lymphedema in this population. This critical difference in breast cancer related lymphedema incidence suggests that preoperative non-linear anatomy may be an independent risk factor for lymphedema.

There are known risk factors for breast cancer-related lymphedema, including (1) axillary lymph node dissection, (2) regional lymph node radiation (RLNR), and (3) high BMI [23, 25–27]. Other debated risk factors include (1) taxane-based chemotherapy, (2) increasing age, and (3) modified radical mastectomy [28–30]. Our findings suggest that prior trauma or invasive procedures to the ipsilateral arm or axillary region is an important additional risk factor for the development of breast cancer-related lymphedema. Prior trauma could incite lymphatic dysfunction that can be further perturbed with direct mechanical force or manipulation during breast cancer procedures. Further research is required to investigate the coorlation of these traumas to the lymphosomes with resultant non-linear lymaphatic anatomy [19]. These results seem to suggest that the presence of non-linear lymphatic anatomy, regardless of the cause or prior trauma history, may be a risk factor for breast cancer-related lymphedema.

These conclusions align with existing literature stating that non-linear patterns represent dysfunctional lymphatic flow [15, 16]. Furthermore, Buchan et. al found that even in patients with pre-operative linear lymphatic anatomy, lymphovenous bypass during ALND may result in changes to the lymphatic anatomy in select patients’ post-operative ICG readings [31]. The authors of this article postulate that these patients may have had risk factors such as minor trauma to the limb that could have lowered the threshould to incite post operative lymphatic flow changes. This potentially supports the theory this paper presents as trauma to the limb of interest could be a risk factor for lymphatic flow disruption.

Globally, patient accessibility to a surgeon trained to perform lymphatic surgery is limited. While lymphedema has gained increasing recognition as a major issue faced by one in five breast cancer survivors, there remains a critical deficit of trained microsurgeons able to perform risk-reducing ILR to meet the needs of these patients [32–34]. Therefore, research focusing on non-invasive, preventative approaches for lymphedema is imperative to target breast cancer patients without access to a lymphatic surgeon or insurance coverage for ILR. Furthermore, continuing collaboration within the fields of plastic surgery, breast surgery, oncology, radiology, and physical therapy is necessary to decrease the risk of lymphedema in this patient population.

Our results suggest that prophylactic interventions may benefit patients with preoperative non-linear lymphatic anatomy, as they appear to be at high risk for lymphedema development. At our Lymphatic Center, patients presenting with non-linear lymphatic anatomy begin prophylactic compression therapy of the affected limb immediately after ALND and ILR. This protocol aims to help target and prevent lymphedema for high-risk patients. The use of compression garments immediately after ALND has been found to significantly decrease the incidence of lymphedema in breast cancer patients, though it is well documented that compression garments have detrimental effects on patient quality of life and compliance levels can vary [35–37]. In institutions where ICG lymphangiography or ILR may not be available, our findings suggest that patients with previous trauma to the limb or those with a large number of nodes removed in a prior SNLB may particularly benefit from early use of compression garments prophylactically. Additionally, diligent follow-up and monitoring can aid in early detection of lymphedema, allowing for earlier intervention.

### Limitations

This study must be interpreted in the context of several limitations. As non-linear lymphatic anatomy on pre-operative ICG lymphography is an uncommon phenomenon, the statistical analysis is underpowered. Nevertheless, our results quantitively demonstrate an association between lymphedema development and non-linear lymphatic anatomy. Our team anticipates a steady growth in the number of patients with non-linear findings, as there is an increasing volume of patients being offered ILR at our institution and greater opportunities for detection through longer follow-up.

## Conclusion

Non-linear lymphatic anatomy on preoperative ICG lymphography appears to be a risk factor for the development of breast cancer related lymphedema. Patients with non-linear anatomy tended to have a history of trauma, procedures, or injury to the ipsilateral arm. Additionally, these patients were more likely to have a larger burden of sentinel lymph nodes excised prior to ALND. Guided by these findings, we have begun to prophylactically prescribe compression garments immediately postoperatively to patients with non-linear lymphatic anatomy on preoperative ICG lymphography in an effort to target and prevent lymphedema development in this high-risk population.

## Data Availability

N/A.
